# Fangs of the Rattlesnake

**Published:** 1883-04

**Authors:** 


Fangs of the Rattlesnake.—At the January meeting of the Philadelphia Academy of Natural Sciences, Dr. Leidy exhibited a series of fangs taken from a rattlesnake fifty-two inches in length. The rapidity with which the functional fangs are reproduced was shown by the presence, on each side of the jaw, of five fangs in varying degrees of development, so placed as to replace those which are lost.

